# Immunohistochemical detection of chlamydia trachomatis in sexually transmitted infectious proctitis

**DOI:** 10.1186/s12876-022-02233-w

**Published:** 2022-04-08

**Authors:** Fernando Arévalo, Soledad Rayme, Fiorella Zurita, Rocio Ramírez, David Franco, Pedro Montes, Jaime Fustamante, Eduardo Monge

**Affiliations:** 1 Hospital Nacional Daniel A Carrión, Callao, Bellavista Peru; 2 Universidad Nacional Mayor de San Marcos, Lima, Peru; 3 Hospital Edmundo Rebagliatti, Lima, Peru; 4 Universidad Particular Cayetano Heredia, Lima, Peru

**Keywords:** Sexually transmitted infectious proctitis, Chlamydia, Pathology, HIV, Immunohistochemistry

## Abstract

**Introduction:**

Since 2003, a progressive increase in sexually transmitted infections (STI), presented as proctitis, has been described in homosexual men. In 2013 Arnold et al. described microscopic features that enable pathologists to formulate a histological diagnosis of STI related proctitis. The aim of this study is to identify the presence of Chlamydia trachomatis by immunohistochemistry in a group of patients with male to male sexual activity and pathology compatible with STI proctitis.

**Methods:**

Cross-sectional study. The study included 54 patients with risky sexual activity and histopathology compatible with STI-proctitis according to Arnold´s recommendations. The Chlamydia trachomatis identification was carried out retrospectively on paraffin blocks using mouse monoclonal antibodies from Santa Cruz biotechnology.

**Results:**

all patients were young men with male to male sexual activity, 69% were positive for HIV. The most common endoscopic presentation was rectal ulcer (61%). Basal lymphoplasmacytic inflammation and mild crypt distortion were the most common histological findings. The immunohistochemical study identified positivity for Chlamydia trachomatis in 40% (18 of 45 tested) of STI proctitis cases.

**Discussion:**

The epidemiological and endoscopic characteristics of the patients studied are similar to those previously reported. In accordance with Arnold et al., the most common histological findings were (a) mild distortion of the crypts; (b) dense and basal lymphoplasmacytic infiltrate and (c) scarcity of eosinophils. The positivity of chlamydia trachomatis in immunohistochemistry was lower than others studies that used PCR for this purpose. We did not find similar published studies to compare our results.

**Conclusions:**

In summary, 54 cases of patients with STI related proctitis are presented, all of them with distinctive histological characteristics and third of the cases tested positive by IHC for Chlamydia trachomatis.

**Supplementary Information:**

The online version contains supplementary material available at 10.1186/s12876-022-02233-w.

## Introduction

Since 2003 an increase in sexually transmitted infectious (STI) has been reported mostly in men having sex with men (MSM) and HIV positive [1, 2]. In this population STI presents as proctitis and is related to microorganisms such as Treponema pallidum (syphilis), gonococcus, herpes virus, and Chlamydia trachomatis particularly the serotypes L1, L2 and L3 that can lead to lymphogranuloma venereum [3]. Usually a specific type of proctitis shows distinctive endoscopic or histological features that make it easily recognizable; however, this is not the case with STIs related proctitis which show heterogeneous features. In fact, STI related proctitis can mimic a rectal neoplasia, ulcerative colitis and even rectal tuberculosis [4], hence it could easily be overlooked or misdiagnosed. Furthermore, if the diagnosis is suspected, identifying the infectious agent is uncommon. The final diagnosis of these infections requires cultures, serological studies or DNA amplification tests, whereas the serological test in the case of chlamydia do not discriminate an old infection from a recent one [4] and the molecular tests are not widely available. Consequently, STI proctitis usually is managed with empirical therapy [5].

Recently Arnold et al. have identified some histological features that support the diagnosis of STI related proctitis in microscopic evaluation [2, 6]; however, specific bacteria detection is not possible with conventional histology staining and the use of immunohistochemistry is required to identify infectious antigens of microorganisms such as Chlamydia trachomatis [7] or Treponema pallidum [8]. Moreover it should be noted that there is currently scarce information on the use of immunohistochemistry in STI related proctitis.

There has been an increase of STI related proctitis in our institution. Initially, some of these cases were mistaken for malignant neoplasms or rectal tuberculosis. The histology, clinical course and medical records suggested an infectious etiology. However, most of them lacked complementary studies that helped identify the specific infectious agent such as chlamydia trachomatis, whose diagnostic tests based on DNA amplification are not available for the majority of our population. The aim of this study is to identify the presence of chlamydia by immunohistochemistry in a group of patients with risky sexual behavior and pathology compatible with STI related proctitis and also to describe the endoscopic and histological features of this proctitis during the 2015–2020 period.

## Methods

Cross-sectional descriptive study.

We included patients between 2015 and 2020 with the following inclusion criteria:

18 years old or older, men with male to male sexual activity (MSM), Rectal biopsies consistent with STI related proctitis

A positive histological biopsy was considered if it fulfilled the following criteria: (1) Lymphoplasmocityc inflammation in mucosa and/or submucosa; (2) Mild architectural distortion (includes crypt enlargement, dilation or branching crypts); (3) Absence of tissue eosinophilia (less than 20 eosinophils HPF). Patients with Inflammatory bowel disease, Colonic Tuberculosis and rectal neoplasms were excluded.

The clinical information was collected from medical records from our hospital. Descriptive statistics were used to summarize the data.

Paraffin-embedded tissue blocks were obtained from pathology storage of our hospital. Tissue sections were stained with hematoxylin–eosin (H&E). The microscopic evaluation was performed using a light microscope from Leica manufacturer: model trinocular BA310E and histological features such as basal inflammation, crypts architecture, eosinophils count, neutrophils, vascular damage, lymphoid follicles and granulomas were assessed.

The immunohistochemical study was performed with a specific mouse IgG2a monoclonal antibody to chlamydia trachomatis as the primary antibody from Santa Cruz biotechnology. Sections of 1.5–2 microns were obtained from the paraffin blocks, placed on positively charged glass slides and then rehydrated with a graded series of ethanol and rinsed with distilled water. The sections were incubated with the primary antibody using a 1: 800 dilution for 20 min at room temperature and then, with the secondary antibody for another 20 min. Diaminobenzidine was used as a chromogen. A sample from a case with a known diagnosis of chlamydia trachomatis infection was used as a positive control. C trachomatis-positive sample was defined by the presence of strongly positive golden brown intracytoplasmic granules.

## Results

A total of 54 STI related proctitis cases from 2015 to 2020 were selected. All of them were male with an age average of 32,4 years, 69% were HIV (+) but only a third were receiving highly active antiretroviral therapy (HAART). Venereal disease research laboratory (VDRL) test was positive in 14 out of 22 patients tested, 7 of them had confirmatory treponema tests.

**Fig. 1:**
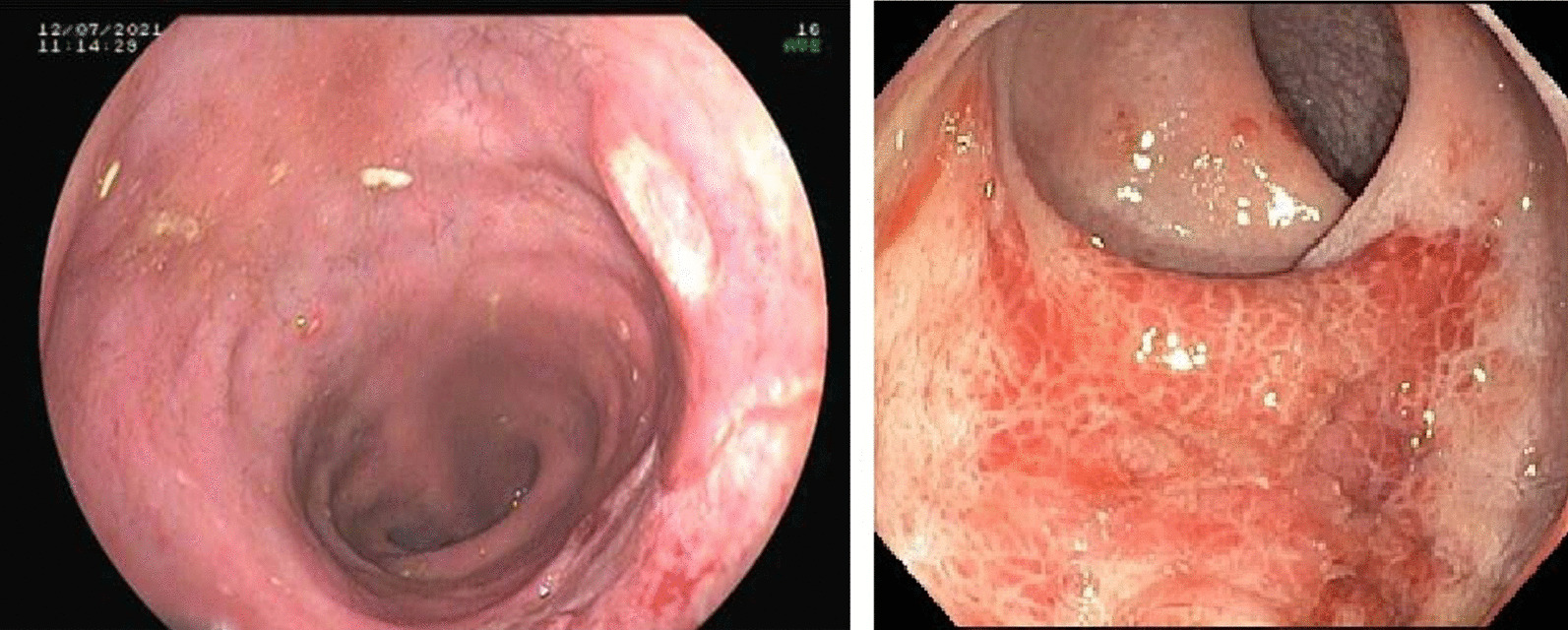
Endoscopic characteristics in 02 cases of rectal compromise. Left picture shows a well-defined ulcer covered with white fibrin. Righ picture depicts an edematous and erythematous ill-defined area with erosions

Compromise was limited to the rectum in all patients examined. The endoscopic features are listed in Table 1. In those with ulcerated lesions, 26/33 had multiple ulcers. Concomitant condylomatosis was reported in 6 and anal fissures were found in 4 patients. The Fig. 1 showed some of these endoscopic appearances.

**Table 1:** Frequency of endoscopic appearance of STI Proctitis (2015–2020)

	Number	%
Ulcer	33	61.1
Erythema and loss of vascular pattern	15	27.8
Mass or tumor	6	11.1
Total	54	100

All cases displayed the features set as criteria to be considered as a STI related proctitis case which include basal lymphoplasmacytic inflammation, mild crypt distortion and few eosinophils with an average of 3 eosinophils per high power field. Additionally, other microscopic characteristics were studied and are detailed in Table 2.

**Table 2:** Frequency of microscopic features of STI proctitis (2015–2020)

	Number	%
Basal lymphoplasmacytic inflammation	54	100
Submucosal involvement	19	35.2
Branched Crypts	19	35.2
Neutrophils in lamina propria	51	94.4
Cryptitis	28	51.8
Incomplete granulomas	03	5.5
Lymphoid follicles	16	29.6
Endothelitis	16	29.6
Total	54	100

**Fig. 2:**
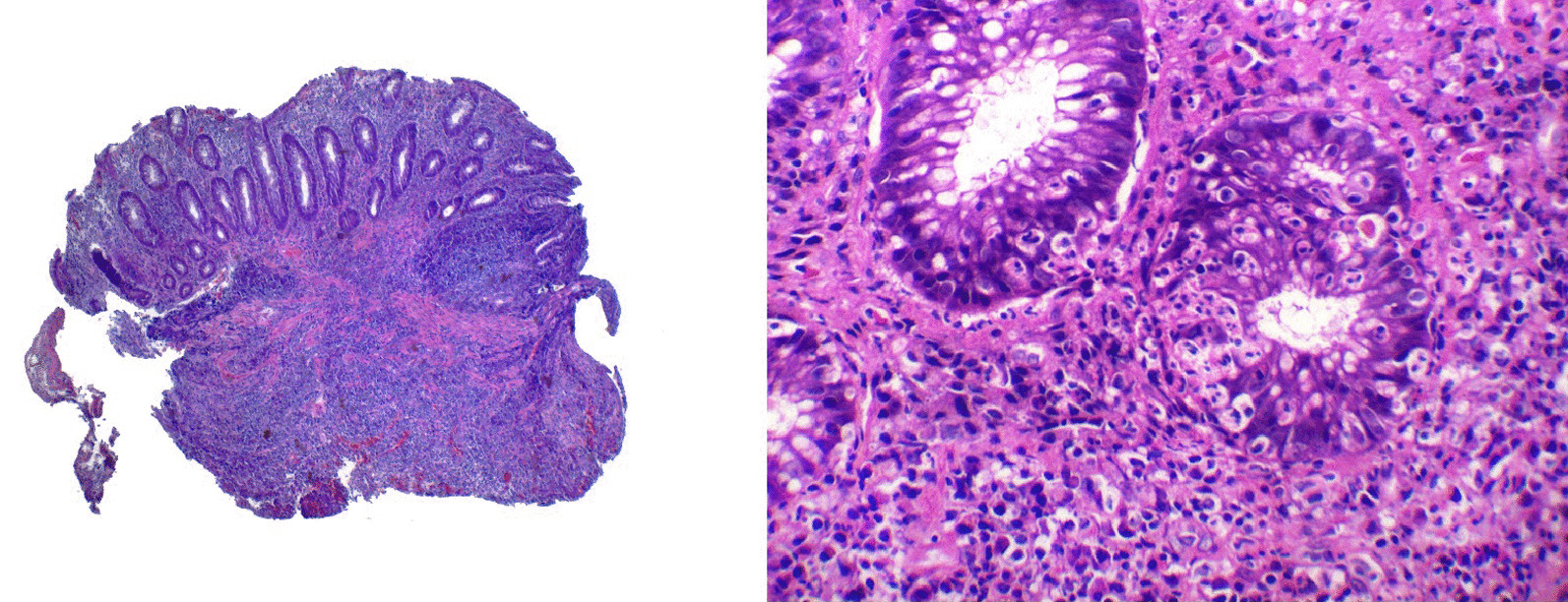
Left, submucosal lymphoplasmacytic infiltrate, note the elongated crypts without branching and a lymphoid follicle. HE 4X. Right: basal lymphoplasmacytic infiltrate and two glands with intraepithelial leucocytes (cryptitis). 10X HE

**Fig. 3:**
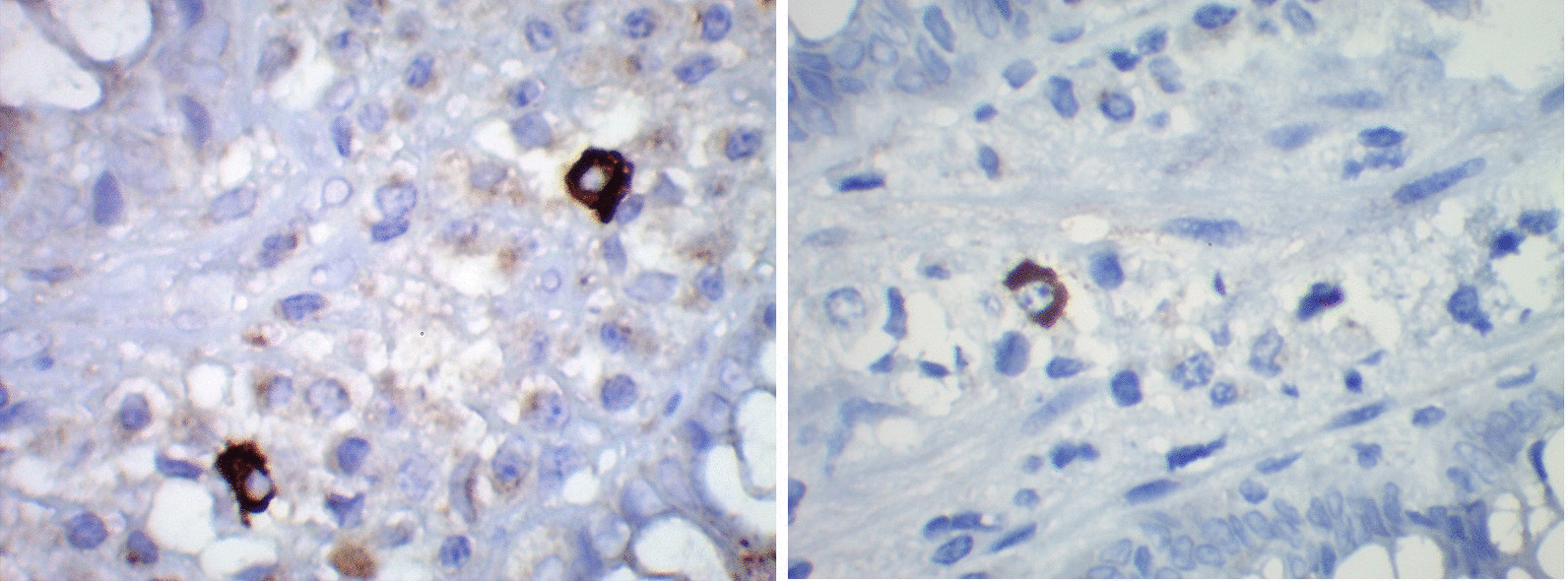
Right and left pictures show macrophages with intense brown staining for Chlamydia trachomatis immunohistochemistry. Immunoperoxidase-diaminobenzidine. 40X

The immunohistochemical study showed the presence of Chlamydia trachomatis in 18 cases, out of 45 tested (40%) and out of total of 54 cases (33%). Positivity was observed as an intracytoplasmic brown staining in macrophages in the stroma of the rectal mucosa. It is worth mentioning that the study was done in 45 cases out of 54 due to the remaining 09 cases the sample from the paraffin block was insufficient to perform the test. Figure 2 shows microscopic photos of our cases, on the right the characteristic histology and on the left the predominantly lymphoplasmacytic infiltrate. Microscopic photos of cells that tested positive for Chlamydia trachomatis infection are shown in Fig. 3.

## Discussion

Recently STI proctitis has gained worldwide attention. This study reports 54 cases of this proctitis in young homosexual men with an average age of 32 years. These findings are in agreement with previous studies, only age is slightly lower compared to other publications, which reported an average of approximately 43 years [9, 10]. These young patients typically present anal pain, tenesmus, constipation, and rectal bleeding [11]. In our study it was not possible to estimate the prevalence of these symptoms due to the lack of systematized information in the medical records, a problem that is quite common in our country.

The coexistence of HIV in patients with STI proctitis has been reported in many publications. Indeed, 70% of cases are considered to be HIV [12] positive. In the present study a similar rate of 69% of HIV positive patients was found. However it must be noted that there was a lack of HIV testing in 13 cases.

The mechanism underlying the association between HIV and STI proctitis is still unknown. It does not seem to be an opportunistic infection because most cases are HIV positive patients who receive antiretroviral treatment (HAART). This could be verified in 20% of our HIV positive cases. Infection with an STD has been considered to enhance HIV transmission, which should be of concern in HIV-negative patients. It is worthy to mention that some HIV positive patients showed lack of interest to avoid infecting their partner, even though they were aware of their contagious disease.

There is a wide range of endoscopic findings in STI proctitis and the most common finding is the ulcerated lesion [2]. In our study more than half of the cases presented as multiple or single ulcers. Garg [13] reported a case of Chlamydia related proctitis described as multiple discontinuous ulcers in the rectum. In Madrid, López-Vicente [14], reported 04 cases of Chlamydia also described as ulcers. Likewise, Arnold reported rectal ulcers as the most frequent presentation of STD proctitis in 7 of their 10 reported cases. Gopal [10] also reported rectal ulcer as the most frequent presentation in 3 of their 4 published cases. The ulcers of our patients were mostly multiple and 04 were suspicious for malignancy but none required surgical management.

The mass or pseudo tumor type lesion is another interesting but unusual endoscopic presentation. Zhao [15] in China reported the case of a patient with rectal syphilis with a mass that occupied the entire circumference of the rectal wall, the diagnosis in this case avoided unnecessary surgery. Taylor [16] in the United Kingdom published a case of Lymphogranuloma venereum described as a friable mass that occupied the entire rectal circumference. Dhawan [12], reported a case of Chlamydia proctitis described as a single proliferative ulcer mass in the rectum. Gopal [10] reported a case of syphilis as an ulcerated multilobed mass highly suspicious of malignancy. Garcia [17] in Spain reported a case associated with Chlamydia with a multilobular and ulcerated pseudo-tumor aspect in the anorectal region. We found 6 cases (11%) described as masses or stenosing lesions, none of them required surgery.

According to Arnold [2], the main histological findings for the diagnosis of STI proctitis are: (a) mild distortion of the crypts; (b) dense and basal lymphoplasmacytic infiltrate and (c) scarcity of eosinophils.

In this study all cases showed these 3 features. The crypts were elongated and dilated in all cases and only occasionally focal branched (30%). Basal lymphoplasmacytic inflammation was usually severe and extending into submucosa in a third of cases. Perivascular plasmacytic infiltrate and endothelitis, which are described mainly in syphilis, were also found in a third of cases [18]. A normal average of 2.5 eosinophils by high—power field was found. These histological features could aid to discriminate from ulcerative colitis which is characterized by a striking architecture distortion with many branched crypts, unaffected submucosa and usually a rise in the number of eosinophils.

Neutrophils could be observed in all patients, cryptitis and microabscess was also found in some of them. According to different authors, the presence of neutrophils is predictive of chlamydial infection [16, 19]. Arnold et al. reported cryptitis in 80% of cases of STI proctitis. Levy [20] found microabscess in 58% of cases of lymphogranuloma venereum. In this regard, our findings are in accordance with the recommendation that neutrophilic activity does not help distinguish STI related proctitis from ulcerative colitis which also shows neutrophilic infiltrate, cryptitis and microabscess.

In 03 cases, clusters of macrophages with incomplete granulomas could be identified. Granulomas have been described as part of inflammatory response associated with syphilis and chlamydia [10, 21]. However, we did not find studies that estimate the prevalence of this finding in STI related proctitis. Sony et al. [19] found granulomas in 5 of 12 cases of chlamydia trachomatis. Tuberculosis is an important pathology to rule out in these cases. In fact, 2 of our cases were first studied as tuberculosis. The appearance of the granuloma is a helpful feature in distinguishing tuberculosis from STI related proctitis, which is deep and accompanied by caseous necrosis and giant cells in tuberculosis, whereas chlamydia or syphilis infection exhibit smaller granulomas and are devoid of caseous necrosis.

Definitive diagnosis of Chlamydia trachomatis requires culture or PCR. The culture could be difficult to obtain and has a low sensitivity [4]. At present, nucleic acid amplification test represents the gold standard for the diagnosis of Chlamydia. Even more, this technique through genotyping can identify the specific chlamydia serovars L1, L2 and L3 [12]. In this regard it has been established that L2 serovar has been the most frequently serovar found in recent epidemic outbreaks [22]. None of these complementary tests were found in our patients medical records. Thus, our study required immunohistochemistry study that helped us to identify retrospectively chlamydia infection.

Immunohistochemical study for Chlamydia trachomatis has been used sporadically and mainly in genitourinary pathology. In this regard, Chlamydia has been identified in cystitis [23] chronic endometritis [7] and placentas [24] of women with a history of recurrent miscarriage. However, in gastrointestinal pathology there is scarce information about this topic. In 2016 Van kruninjen [21] using immunohistochemistry showed positive staining for Chlamydia trachomatis in the cytoplasm of macrophages in submucosa and serosa of colon and ileum of 5 of 19 patients with crohn's disease. Further, Borel [25] using real time PCR detected chlamydia in the colon of 4 patients, 2 of them also stained positive for Chlamydia using immunohistochemistry.

In our study a third of STI related proctitis cases were positive for chlamydia using immunohistochemistry. We did not find similar published studies to compare our results. However, there is an investigation in Tel Aviv carried out using PCR [20] in 17 patients with STI related Proctitis, which demonstrated chlamydia infection in 82% of their cases. This difference highlights the greater sensitivity of molecular tests in relation to immunohistochemistry, but it also shows the importance of Chlamydia in the etiology of these lesions. It should be mentioned that a subset of 09 of Chlamydia positive cases also tested positive for syphilis by serology, this co-infection is not surprising given both germs share the same risk factors.

## Conclusions

In summary, 54 cases of patients with STI related proctitis are presented, all of them with distinctive histological characteristics and third of the cases tested positive by IHC for Chlamydia trachomatis.

## Supplementary Information

**Additional file 1: Proctits ITS.** Submucosal lymphoplasmacytic infiltrate, note the elongated and dilated crypts without branching and a lymphoid follicle.

**Additional file 2: Proctits ITS.** Submucosal lymphoplasmacytic infiltrate, note the elongated and dilated crypts without branching.

**Additional file 3: chlamydia immunohistochemistry:** the picture depicts show macrophages with intense brown staining for Chlamydia trachomatis immunohistochemistry. Immunoperoxidase-diaminobenzidine.

**Additional file 4: chlamydia immunohistochemistry:** the picture depicts show macrophages with intense brown staining for Chlamydia trachomatis immunohistochemistry. Immunoperoxidase-diaminobenzidine.

**Additional file 5: chlamydia immunohistochemistry:** the picture depicts show macrophages with intense brown staining for Chlamydia trachomatis immunohistochemistry. Immunoperoxidase-diaminobenzidine.

**Additional file 6: Proctits ITS.** Submucosal lymphoplasmacytic infiltrate, note the elongated and dilated crypts without branching.

**Additional file 7: Proctits ITS.** Submucosal lymphoplasmacytic infiltrate, note the elongated and dilated crypts without branching.

**Additional file 8: RAW DATA TABLE.** Histology and endoscopic findings Proctitis ITS. 2015-2020 Lima.

## Data Availability

All data generated or analysed during this study are included in this published article and its supplementary information files.
