# Supplementary figures and images for: Immunohistochemical detection of chlamydia trachomatis in sexually transmitted infectious proctitis
Source: BMC Gastroenterol. 2022 Apr 8;22:171. doi: 10.1186/s12876-022-02233-w (PMC8991980; doi:10.1186/s12876-022-02233-w)

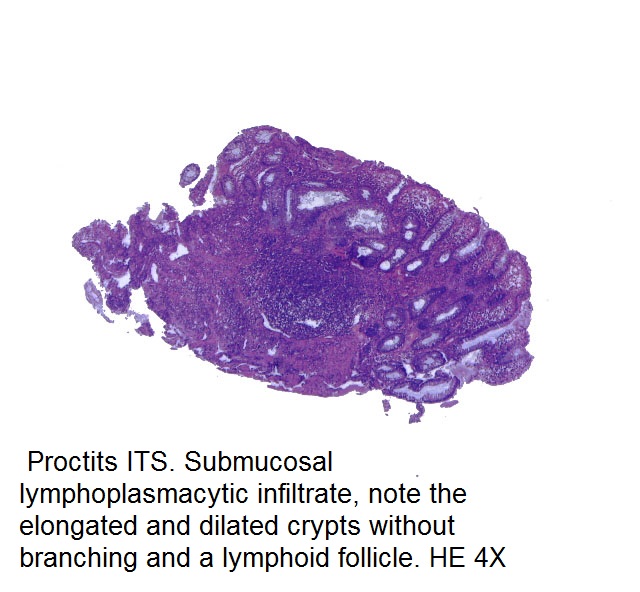

Supplement: Supplementary file 1 — Additional file 1: Proctits ITS. Submucosal lymphoplasmacytic infiltrate, note the elongated and dilated crypts without branching and a lymphoid follicle. [file 12876_2022_2233_MOESM1_ESM.jpg]

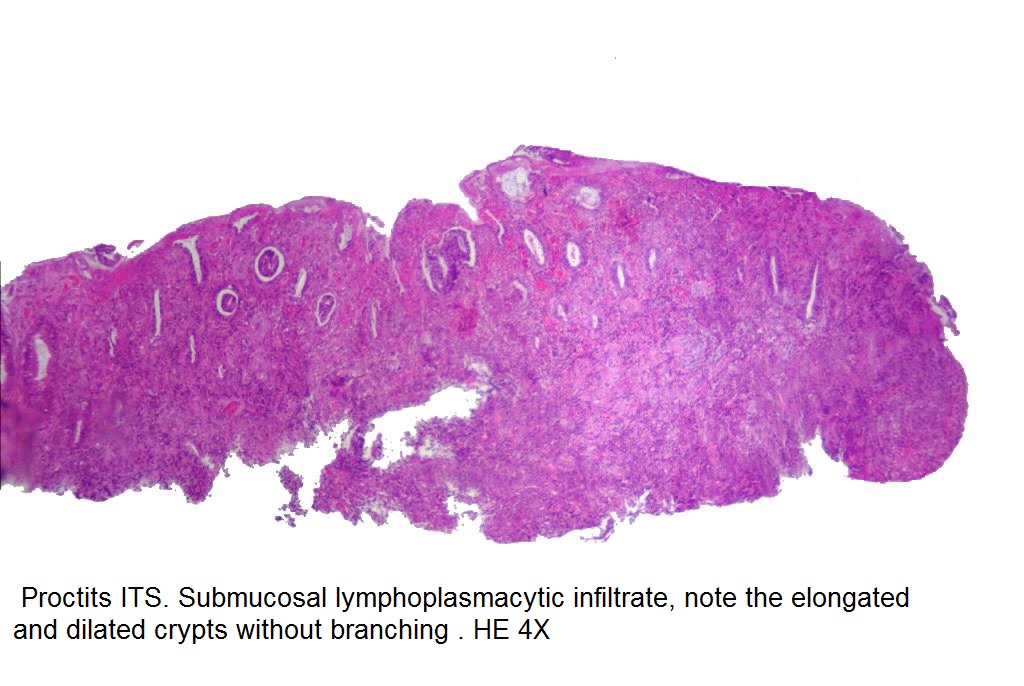

Supplement: Supplementary file 2 — Additional file 2: Proctits ITS. Submucosal lymphoplasmacytic infiltrate, note the elongated and dilated crypts without branching. [file 12876_2022_2233_MOESM2_ESM.jpg]

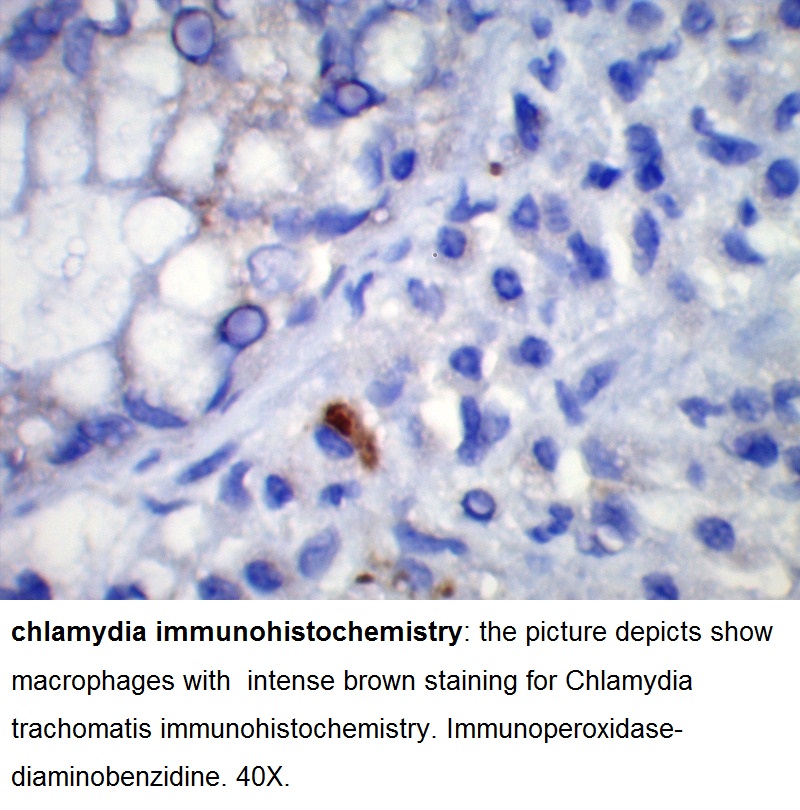

Supplement: Supplementary file 3 — Additional file 3: chlamydia immunohistochemistry: the picture depicts show macrophages with intense brown staining for Chlamydia trachomatis immunohistochemistry. Immunoperoxidase-diaminobenzidine. [file 12876_2022_2233_MOESM3_ESM.jpg]

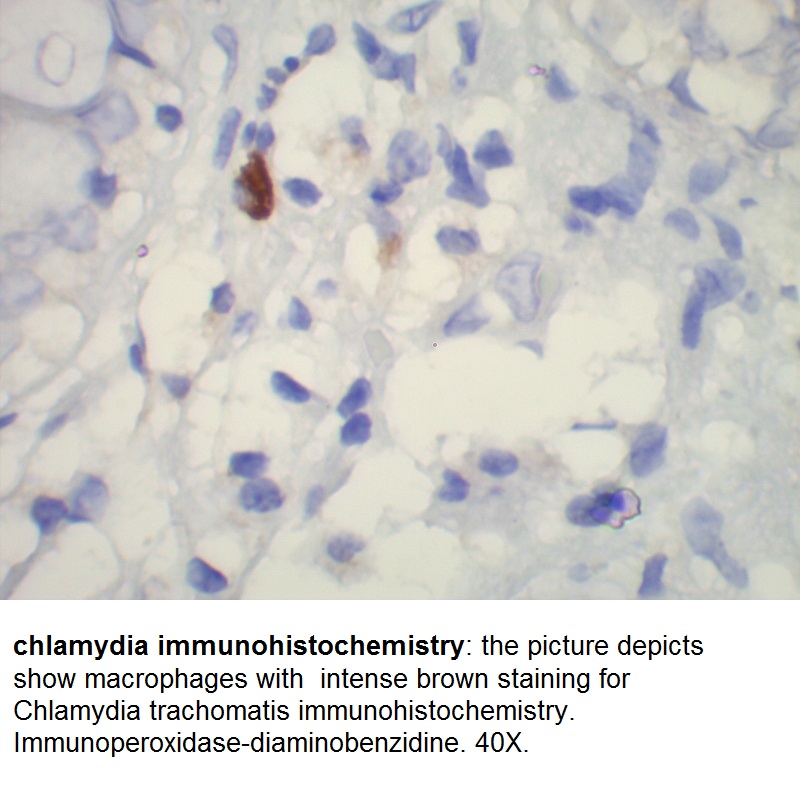

Supplement: Supplementary file 4 — Additional file 4: chlamydia immunohistochemistry: the picture depicts show macrophages with intense brown staining for Chlamydia trachomatis immunohistochemistry. Immunoperoxidase-diaminobenzidine. [file 12876_2022_2233_MOESM4_ESM.jpg]

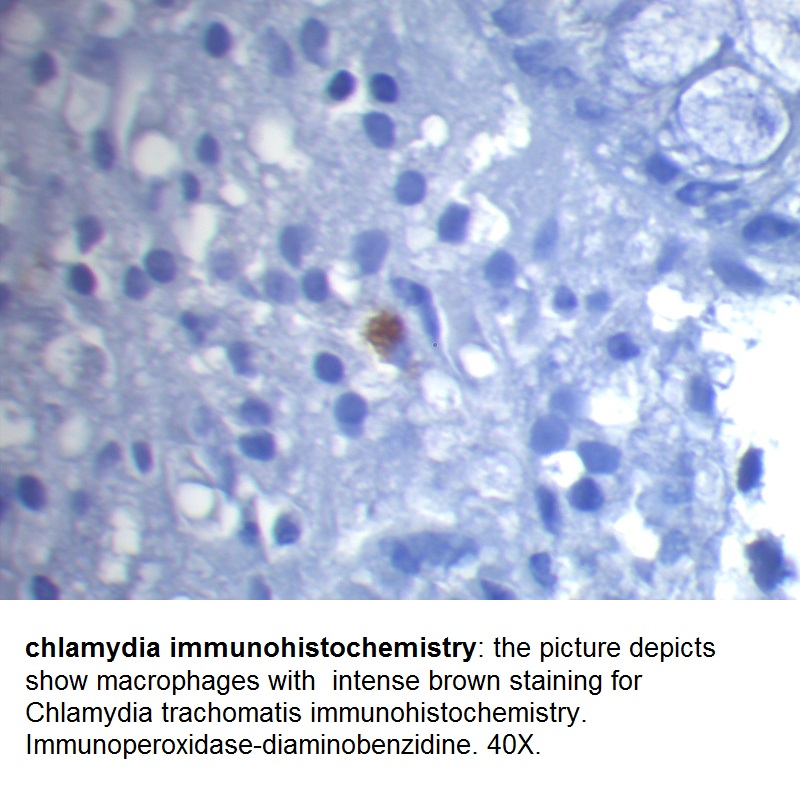

Supplement: Supplementary file 5 — Additional file 5: chlamydia immunohistochemistry: the picture depicts show macrophages with intense brown staining for Chlamydia trachomatis immunohistochemistry. Immunoperoxidase-diaminobenzidine. [file 12876_2022_2233_MOESM5_ESM.jpg]

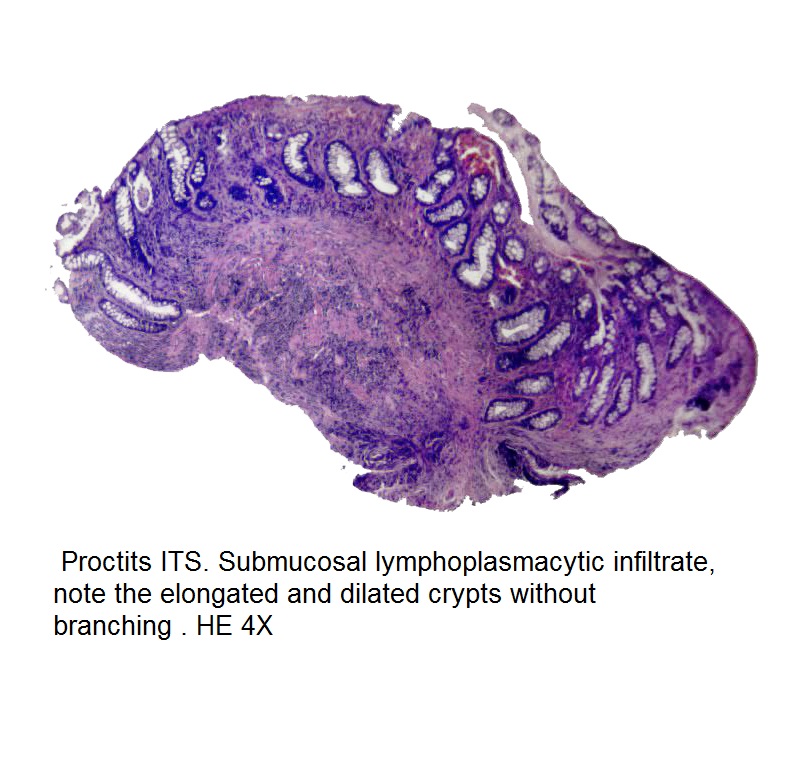

Supplement: Supplementary file 6 — Additional file 6: Proctits ITS. Submucosal lymphoplasmacytic infiltrate, note the elongated and dilated crypts without branching. [file 12876_2022_2233_MOESM6_ESM.jpg]
