# Supplementary material for: Immunohistochemical detection of chlamydia trachomatis in sexually transmitted infectious proctitis
Source: BMC Gastroenterol. 2022 Apr 8;22:171. doi: 10.1186/s12876-022-02233-w (PMC8991980; doi:10.1186/s12876-022-02233-w)
Supplement: Supplementary file 8 — Additional file 8: RAW DATA TABLE. Histology and endoscopic findings Proctitis ITS. 2015-2020 Lima. [file 12876_2022_2233_MOESM8_ESM.pdf]

RAW DATA TABLE. Histology and endoscopic findings Proctitis ITS. 2015-2020 Lima. Peru

code	HC	age	gender	endoscopy lesion	HIV	VDRL	treponemic specific tests	branched crypts	dilated crypts	inflammation	neutrophils	cryptitis	eosinophils per HPF	lymp follicles	IHC chlamydia
15-4282	851275	38	M	multiple ulcers	HIV POSITIVE	VDRL POSITIVE	POSITIVO	branched crypts presents	dilated crypts present	inflamación basal presente	neutrophils present	cryptitis present	0	l.p.absent	CHLAMYDIA IHC NEGATIVE
15-7002	1276621	38	M	multiple ulcers	HIV POSITIVE	VDRL POSITIVE	NOT STATED	branched crypt absent	dilated crypts present	inflamación basal presente	neutrophils present	cryptitis present	4	lymph follicles present	CHLAMYDIA IHC NEGATIVE
15-8172	811034	44	M	tumor or mass	HIV NEGATIVE	NOT STATED	NOT STATED	branched crypt absent	dilated crypts present	basal inflammation	neutrophils present	cryptitis present	4	l.p.absent	CHLAMYDIA IHC NEGATIVE
15-8890	1444905	27	M	multiple ulcers	HIV POSITIVE	VDRL POSITIVE	NOT STATED	branched crypts presents	dilated crypts present	inflamación basal presente	neutrophils present	cryptitis present	3	l.p.absent	CHLAMYDIA IHC POSITIVE
16-0185		28	M	tumor or mass	HIV POSITIVE	VDRL NEGATIVE	NOT STATED	branched crypt absent	dilated crypts present	basal inflammation	neutrophils present	ABSENT	2	l.p.absent	NOT ASSSESED
16-3613	972783	31	M	multiple ulcers	HIV POSITIVE	VDRL POSITIVE	POSITIVO	branched crypt absent	dilated crypts present	inflamacion basal presente	neutrophils present	ABSENT	4	l.p.absent	CHLAMYDIA IHC POSITIVE
17-0586	1561541	22	M	ulcers and polyps	HIV POSITIVE	NOT STATED	NOT STATED	branched crypt absent	dilated crypts present	basal inflammation	ABSENT	ABSENT	0	lymph follicles present	NOT ASSSESED
17-4160	1156247	43	M	multiple ulcers	HIV POSITIVE	VDRL NEGATIVE	NOT STATED	branched crypt absent	dilated crypts present	basal inflammation	neutrophils present	cryptitis present	3	l.p.absent	NOT ASSSESED
17-6828		25	M	tumor or mass	HIV POSITIVE	NOT STATED	NOT STATED	branched crypts presents	dilated crypts present	absent	neutrophils present	cryptitis present	3	l.p.absent	CHLAMYDIA IHC NEGATIVE
18-2607	500134	30	M	multiple ulcers	HIV POSITIVE	VDRL POSITIVE	NOT STATED	branched crypts presents	dilated crypts present	inflamacion basal presente	neutrophils present	cryptitis present	0	l.p.absent	CHLAMYDIA IHC NEGATIVE
18-3314-1	pARTICULAR pero es del carrion	21	m	multiple ulcers	HIV NEGATIVE	VDRL POSITIVE	NEGATIVO	branched crypt absent	ABSENT	inflamación basal ausente	neutrophils present	cryptitis present	3	lymph follicles present	CHLAMYDIA IHC POSITIVE
18-5528	particular	25	M	tumor or mass	HIV POSITIVE	VDRL POSITIVE	POSITIVO	branched crypts presents	dilated crypts present	inflamación basal presente	neutrophils present	cryptitis present	1	l.p.absent	CHLAMYDIA IHC POSITIVE
18-6893	1561546	25	M	multiple ulcers	HIV POSITIVE	VDRL POSITIVE	NEGATIVO	branched crypt absent	dilated crypts present	inflamación basal presente con granuloma incompleto	neutrophils present	ABSENT	10	l.p.absent	CHLAMYDIA IHC NEGATIVE
18-7240	765352	35	M	ulcers and polyps	HIV POSITIVE	NOT STATED	NOT STATED	branched crypts presents	dilated crypts present	basal inflammation	neutrophils present	cryptitis present	0	lymph follicles present	CHLAMYDIA IHC POSITIVE
18-9283	309470	47	M	proctitis	HIV POSITIVE	VDRL POSITIVE	POSITIVO	branched crypt absent	dilated crypts present	inflamación basal presente	neutrophils present	cryptitis present	0	l.p.absent	CHLAMYDIA IHC POSITIVE
19-0991-2	821547	50	M	ulcers and polyps	HIV POSITIVE	VDRL NEGATIVE	NOT STATED	branched crypt absent	dilated crypts present	basal inflammation	neutrophils present	cryptitis present	3	lymph follicles present	CHLAMYDIA IHC POSITIVE
19-2713		25	M	ulcers and polyps	HIV POSITIVE	NOT STATED	NOT STATED	branched crypt absent	ABSENT	absent	neutrophils present	cryptitis present	2	l.p.absent	NOT ASSSESED
19-3315	1561546	26	M	multiple ulcers	HIV POSITIVE	VDRL POSITIVE	NEGATIVO	branched crypts presents	dilated crypts present	inflamación basal presente	neutrophils present	ABSENT	5	l.p.absent	CHLAMYDIA IHC POSITIVE
19-3587	1758950	27	M	proctitis	HIV POSITIVE	VDRL POSITIVE	POSITIVO	branched crypt absent	dilated crypts present	inflamación crónica superficial presente y basal leve	ABSENT	ABSENT	0	l.p.absent	CHLAMYDIA IHC POSITIVE
19-4891 de Fuati	PARTICULAR	30	M	proctitis	HIV POSITIVE	VDRL NEGATIVE	NOT STATED	branched crypts presents	dilated crypts present	basal inflammation	neutrophils present	cryptitis present	2	lymph follicles present	CHLAMYDIA IHC NEGATIVE
19-5006	1765670	28	M	ulcers and polyps	NOT STATED	NOT STATED	NOT STATED	branched crypts presents	dilated crypts present	basal inflammation	neutrophils present	cryptitis present	0	lymph follicles present	CHLAMYDIA IHC NEGATIVE
19-5431	1730736	22	M	multiple ulcers	HIV POSITIVE	VDRL NEGATIVE	NOT STATED	branched crypt absent	dilated crypts present	basal inflammation extending into submucosa	neutrophils present	cryptitis present	1	l.p.absent	CHLAMYDIA IHC POSITIVE
19-5624		54	M	multiple ulcers	HIV POSITIVE	NOT STATED	NOT STATED	branched crypts presents	dilated crypts present	basal inflammation	neutrophils present	cryptitis present	0	l.p.absent	CHLAMYDIA IHC NEGATIVE
19-6793	PARTICULAR	37	M	multiple ulcers	HIV NEGATIVE	NOT STATED	NOT STATED	branched crypt absent	dilated crypts present	basal inflammation	neutrophils present	cryptitis present	4	lymph follicles present	CHLAMYDIA IHC POSITIVE
19-6851		37	M	single ulcer	HIV POSITIVE	VDRL POSITIVE	POSITIVO	branched crypts presents	dilated crypts present	inflamacion cronica basal presente	neutrophils present	cryptitis present	2	l.p.absent	CHLAMYDIA IHC POSITIVE
19-8216	PARTICULAR	26	M	multiple ulcers	HIV POSITIVE			branched crypt absent	dilated crypts present	basal inflammation	neutrophils present	cryptitis present	2	l.p.absent	CHLAMYDIA IHC NEGATIVE
20-0412-4		38	M	tumor or mass	HIV POSITIVE	VDRL NEGATIVE		branched crypts presents	dilated crypts present	basal inflammation extending into submucosa and endarteritis	neutrophils present	cryptitis present	3	l.p.absent	NOT ASSSESED
20-0494		18	M	proctitis	NOT STATED	NOT STATED		branched crypt absent	dilated crypts present	basal inflammation	neutrophils present	cryptitis present	1	l.p.absent	NOT ASSSESED
20-0668		29	M	multiple ulcers	HIV POSITIVE	NOT STATED		branched crypt absent	dilated crypts present	basal inflammation	neutrophils present	cryptitis present	1	l.p.absent	NOT ASSSESED
20-1069-2		25	M	multiple ulcers	NOT STATED			branched crypt absent	dilatadas	basal inflammation extending into submucosa and endarteritis	neutrophils present	ABSENT	5	l.p.absent	CHLAMYDIA IHC NEGATIVE
20-2327		50	M	proctitis	HIV POSITIVE	NOT STATED	NOT STATED	branched crypts presents	ABSENT	basal inflammation	neutrophils present	ABSENT	4	lymph follicles present	CHLAMYDIA IHC POSITIVE
20-3414	INKAMAY SALUD	21	M	multiple ulcers	NOT STATED	NOT STATED		branched crypt absent	dilated crypts present	basal inflammation	neutrophils present	ABSENT	0	l.p.absent	CHLAMYDIA IHC POSITIVE
20-4664	SISOL VMT	24	M	proctitis	HIV POSITIVE	NOT STATED		branched crypt absent	dilated crypts present	basal inflammation extending into submucosa and endarteritis	neutrophils present	ABSENT	1	l.p.absent	CHLAMYDIA IHC POSITIVE
20-4760	OMAR CORO	34	M	proctitis	HIV POSITIVE			branched crypt absent	ABSENT	basal inflammation extending into submucosa and endarteritis	neutrophils present	cryptitis present	8	lymph follicles present	CHLAMYDIA IHC NEGATIVE

20-5025	POLICLÍNICO INTERNACIONAL	40	M	tumor or mass	HIV POSITIVE			branched crypt absent	dilated crypts present	basal inflammation with incomplete granuloma	ABSENT	ABSENT	1	l.p.absent	CHLAMYDIA IHC NEGATIVE
20-5729	SISOL VMT	28	M	multiple ulcers	NOT STATED			branched crypt absent	dilatadas	basal inflammation extending into submucosa and incomplete granuloma	neutrophils present	ABSENT	0	l.p.absent	CHLAMYDIA IHC NEGATIVE
20-5936	PARTICULAR	27	M	proctitis	HIV NEGATIVE	VDRL POSITIVE	POSITIVO	branched crypts presents	dilatadas	inflamación con extensión a submucosa y lesion tipo endotelitis y manguitos de celulas plasmaticas	neutrophils present	ABSENT	0	l.p.absent	CHLAMYDIA IHC NEGATIVE
20-6516	SISOL VMT	33	M	proctitis	HIV POSITIVE			branched crypt absent	dilatadas	basal inflammation	neutrophils present	ABSENT	1	lymph follicles present	CHLAMYDIA IHC NEGATIVE
21-0212		32	M	multiple ulcers	HIV POSITIVE			branched crypt absent	dilatadas	basal inflammation	neutrophils present	ABSENT	6	l.p.absent	NOT ASSSESED
21-0229	GASTRO BRASIL	24	M	multiple ulcers	VIH PENDIENTE	NOT STATED	NOT STATED	branched crypt absent	dilatadas	basal inflammation	neutrophils present	cryptitis present	1	lymph follicles present	CHLAMYDIA IHC NEGATIVE
21-0445	GASTRO BRASIL	35	M	proctitis	HIV POSITIVE			branched crypts presents	dilatadas	basal inflammation with endarteritis	neutrophils present	ABSENT	1	lymph follicles present	CHLAMYDIA IHC NEGATIVE
21-0587	SISOLVMT	25	M	single ulcer	HIV POSITIVE	VDRL POSITIVE	NOT STATED	branched crypts presents	dilatadas	inflamación con extension a submucosa focal sin granulomas sin endotelitis	neutrophils present	ABSENT	2	l.p.absent	CHLAMYDIA IHC POSITIVE
21-0751	POL.INTERNACIO	27	M	multiple ulcers	HIV POSITIVE			branched crypt absent	dilatadas	basal inflammation with endarteritis	neutrophils present	cryptitis present	1	l.p.absent	CHLAMYDIA IHC NEGATIVE
21-0859-2	PARTICULAR	47	M	proctitis	HIV POSITIVE	NO CONSIGNADO	positivo	branched crypt absent	dilatadas	basal inflammation extending into submucosa and endarteritis and clusters of macrophages	neutrophils present	criptitis	3	l.p.absent	CHLAMYDIA IHC POSITIVE
21-0976	P.INTERNACIONAL	36	M	single ulcer	VIH PENDIENTE	NOT STATED	NOT STATED	branched crypt absent	dilatadas	basal inflammation	neutrophils present	ABSENT	4	lymph follicles present	CHLAMYDIA IHC NEGATIVE
21-1628	PARTICULAR	27	M	proctitis	NOT STATED	NOT STATED	NOT STATED	branched crypts presents	dilatadas	basal inflammation	neutrophils present	ABSENT	12	l.p.absent	CHLAMYDIA IHC NEGATIVE
21-1796	PARTICULAR	51	M	single ulcer	NOT STATED	NOT STATED	NOT STATED	branched crypt absent	dilatadas	basal inflammation and endarteritis	neutrophils present	ABSENT	0	l.p.absent	CHLAMYDIA IHC NEGATIVE
21-2446	POL.MUNICIPAL	41	M	single ulcer	NOT STATED	NOT STATED	NOT STATED	branched crypts presents	dilatadas	basal inflammation and endarteritis	neutrophils present	ABSENT	2	l.p.absent	CHLAMYDIA IHC NEGATIVE
21-2462	P. INTERNACIONAL	30	M	single ulcer	NOT STATED			branched crypt absent	dilatadas	basal inflammation and endarteritis	neutrophils present	cryptitis present	0	l.p.absent	CHLAMYDIA IHC NEGATIVE
21-2747	P.INTERNACIONAL	26	M	single ulcer	HIV POSITIVE	NOT STATED		branched crypt absent	dilatadas	basal inflammation with clusters of macrophages	neutrophils present	ABSENT	14	lymph follicles present	CHLAMYDIA IHC POSITIVE
21-3232-1	PARTICULAR	24	M	PROCTITIS	HIV POSITIVE	VDRL NEGATIVE	NO	branched crypt absent	dilatadas	basal inflammation and endarteritis	neutrophils present	ABSENT	2	l.p.absent	CHLAMYDIA IHC NEGATIVE
21-3550	OMAR CORO	53	M	PROCTITIS	NOT STATED	NOT STATED	NO	branched crypt absent	dilatadas	basal inflammation	neutrophils present	cryptitis present	2	lymph follicles present	CHLAMYDIA IHC NEGATIVE
21-4074	PARTICULAR	29	M	PROCTITIS	HIV POSITIVE	NOT STATED	NO	branched crypt absent	dilatadas	basal inflammation	neutrophils present	ABSENT	5	l.p.absent	CHLAMYDIA IHC NEGATIVE
21-4954	HNDAC	37	M	single ulcer	HIV POSITIVE	VDRL NEGATIVE		branched crypts presents	dilatadas	basal inflammation extending into submucosa and endarterit	neutrophils present	cryptitis present	4	l.p.absent	CHLAMYDIA IHC NEGATIVE
